# Families' Perception of Cognitive and Emotional Support From Healthcare Professionals Across the Maternal and Newborn Care Continuum

**DOI:** 10.1111/nicc.70337

**Published:** 2026-01-16

**Authors:** Christina Schuler, George Edward Ntow, Wisdom Kudzo Axame, Emmanuel Bansah, Barbara Preusse‐Bleuler, Riccardo E. Pfister, Agbozo Faith

**Affiliations:** ^1^ Institute of Global Health, Faculty of Medicine University of Geneva Geneva Switzerland; ^2^ Institute of Nursing, School of Health Sciences Zurich University of Applied Sciences (ZHAW) Winterthur Switzerland; ^3^ Implementation Science Department Dodowa Health Research Centre Dodowa Ghana; ^4^ Department of Epidemiology and Biostatistics, FN Binka School of Public Health University of Health and Allied Sciences Ho Ghana; ^5^ Neonatal and Paediatric Intensive Care Unit University Hospitals of Geneva and Geneva University Geneva Switzerland; ^6^ Department of Family and Community Health, FN Binka School of Public Health University of Health and Allied Sciences Ho Ghana

**Keywords:** family systems care, family‐centred care, Ghana, involvement, perinatal care

## Abstract

**Background:**

During the perinatal period, women and newborns require high‐quality supportive care. While cognitive and emotional care support is central to family systems care, few quantitative studies have explored this in sub‐Saharan Africa.

**Aim:**

We investigated families' perspectives on the support provided by healthcare professionals during maternal and newborn care, examining the impact of family demographics on their perceived support and identifying unmet support needs.

**Study Design:**

This cross‐sectional survey was conducted in the Hohoe Municipality, Ghana. Participants included high‐risk pregnant, birthing and postnatal women, mothers with small/sick newborns and their family members. Family support was assessed using the Icelandic Family Perceived Support Questionnaire (5‐point Likert; scores: cognitive 5–25, emotional 9–45, overall 14–70). Descriptive and inferential statistics were conducted with STATA. Analysis of open‐ended text elements was done in two cycles, employing open coding and thematic categorisation via NVIVO software.

**Results:**

Participants (*N* = 442) perceived their overall support from healthcare professionals, covering antenatal, labour, neonatal intensive care, postnatal and community‐based primary care, as deficient (mean 45.3 ± 14.2). Emotional support (mean 16.6 ± 5.9) was rated lower than cognitive support (mean 28.6 ± 10.1). The highest rating was assigned to the item regarding taking respite (mean 4.0 ± 1.6). The item on family encouragement to narrate their illness experience, family strength and resilience received the lowest rating (mean 2.5 ± 1.8). Older family members and males felt excluded from care. Families requested consideration of their preferences, such as integrating alternative medicine and spirituality into care plans.

**Conclusion:**

Cognitive support throughout the care continuum was perceived as average and emotional support was even lower. Family systems care guidelines and skills training are needed to strengthen healthcare professionals' communication skills to provide psychologically and emotionally safe support along the perinatal care continuum.

**Relevance to Clinical Practice:**

Family meetings that address previous experiences, provide details regarding illnesses and outline care plans can improve family participation and resilience.

## Impact Statements

- What is known about the topic
  ○ In high‐risk pregnancies or when caring for small and sick newborns, families require strong supportive care, including cognitive and emotional care.
  ○ Perinatal care is often hindered by disrespect and the exclusion of family members from care decisions.
  ○ Through family systems care, healthcare professionals can provide cognitive and emotional support to families throughout the perinatal period.
- What this paper adds
  ○ Shows that families perceive overall support from healthcare professionals across the care continuum as inadequate, with emotional support rated significantly lower than cognitive support.
  ○ Demonstrates the need to improve care experiences and quality in maternal and newborn health along the continuum of care, particularly focusing on emotional supportive care.
  ○ Emphasises the importance of providing timely, easy‐to‐understand information on illnesses to all family members, including male partners and older relatives.

**Abbreviations**
ANC: antenatal care
CHPS: community‐based health planning and services
FSC: family systems care
HCPs: healthcare professionals
ICE‐FPSQ: Iceland‐family perceived support questionnaire
NICU: neonatal intensive care unit

## 1 Introduction

Pregnancy, childbirth and the postpartum phase are significant life events, with the health and well‐being of women, and their newborns closely intertwined [1]. During this vulnerable period, women and newborns require high‐quality care. High‐quality care contributes to the health and well‐being of pregnant women, neonates and their families and includes clinically secure, psychologically and emotionally safe care [2].

The care continuum from pregnancy, childbirth and the postnatal period should be a positive experience for women, newborns and their families. However, maternal and newborn care is often challenged by disrespect and abuse, jeopardising patient rights and negatively affecting maternal and neonatal outcomes [3, 4, 5, 6, 7]. The WHO's Quality of Care Framework and the compendium on respectful maternal and newborn health emphasise delivering a positive experience by effectively communicating, providing respectful and continuing care in a safe environment and offering a human‐rights‐based supportive care approach [1, 2].

Supportive care improves families' ability to cope with illness, strengthens their well‐being and enhances family functioning [8, 9, 10]. Supportive care includes cognitive and emotional support. Cognitive support is given by healthcare professionals (HCPs) through information or education to allow families to handle their illness experience. It refers to strategies, tools or interventions that help individuals understand, process and retain information effectively. Emotional support encompasses the assistance to families in managing the emotional distress related to a family member's condition through empathy, respect and engaged relational listening. It has been shown to increase the well‐being of the whole family [10, 11].

When experiencing health challenges associated with pregnancy, childbirth and the postnatal period, families need cognitive and emotional support to alleviate anxiety and uncertainty. Particularly during high‐risk pregnancies or when having a small and sick newborn, families require supportive care [12]. Since illness affects not only the individual but also their family members, it is essential to provide support to the family unit as a whole [13, 14].

## 2 Theoretical Framework

This study is guided by the concept of Family Systems Care (FSC). FSC is an increasingly recognised approach that represents a shift from individual‐centred care toward relational and systemic models of support, acknowledging the profound impact of illness on the entire family. It is based on the Calgary family assessment and family intervention models (CFAM and CFIM) [15]. These models include systemic interventions such as positive manners toward families, relationship building, drawing of family genograms, therapeutic conversations and provision of commendations on family strength and resources [16]. In FSC, relationship building between HCPs and families is central.

## 3 Justification of Study

While essential, clear communication, emotional support and respectful care for women, newborns and families are infrequently prioritised in research, practice and education despite their affordability [2, 17].

Even though HCPs believe they provide families with adequate support [18], families often report a lack of the needed support [19]. This gap requires evaluation through quantitative and qualitative research that captures families' perceptions of the support they received or wished they had received. To date, few quantitative studies have explored the perceived family support within the sub‐Saharan African context [18, 19, 20]. As this understanding is crucial for improvement of care [21], we investigated this gap by conducting a cross‐sectional study.

## 4 Aims

We aimed to (1) determine families' perceptions of support received by healthcare professionals along the maternal and newborn healthcare continuum in the Hohoe Municipality, Ghana; (2) examine what demographic characteristics influence their cognitive and emotional support scores and (3) assess what care support families felt was lacking.

## 5 Methods

### 5.1 Design and Method

This cross‐sectional survey was conducted between August 2023 and January 2024 in the Hohoe Municipality of the Volta Region in Ghana. The study adhered to the Strengthening the Reporting of Observational Studies in Epidemiology (STROBE) guidelines for articles reporting cross‐sectional studies [22, 23].

### 5.2 Setting

The Hohoe Municipality has a large youth population of 35.9% under the age of 15. A small percentage works in the formal sector, while most are involved in farming and informal trading [24]. Literacy rate in the Volta Region is 83.9% for males and 67.6% for females [25]. A population of at least 200 000 is served by the municipality's healthcare system, which includes the Volta regional hospital, the public health and nutrition unit, and 13 community‐based primary care facilities (including Community‐based Health Planning and Services (CHPS) zones and health centres).

The Volta regional hospital offers obstetric and essential newborn care as well as care for small and sick neonates. The community‐based primary care facilities provide basic Maternal, Newborn and Child Health (MNCH) services and home visits. Our study was conducted at the Volta regional hospital, with facility details described previously [26]. At the VRH Volta regional hospital, family members were allowed to stay near their hospitalised relatives, but no accommodation was provided.

### 5.3 Sample

**FIGURE 1:**
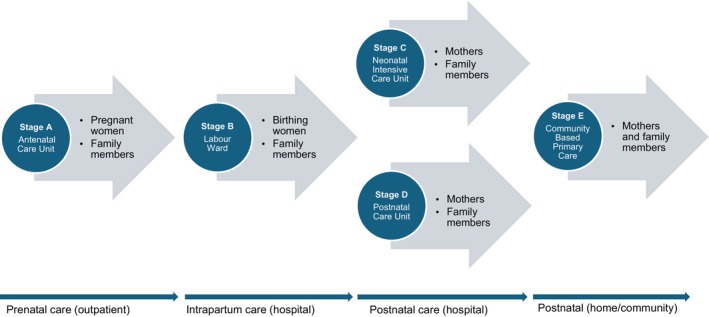
The nine participant groups classified across five stages in the maternal and newborn care continuum.

We surveyed nine participant groups at five different stages in the maternal and newborn care continuum: (A) antenatal care unit (ANC), (B) labour ward, (C) neonatal intensive care unit (NICU), (D) postnatal ward and (E) community‐based primary care facilities (Figure 1). Eligible individuals were consecutively enrolled until a designated quota was reached. In the community‐based primary care setting, data collection was limited to a 2‐month period as quota sampling was impossible. Analysing broader family support members independently of mothers and newborns at each stage of care revealed nine distinct participant groups, as outlined below.

### 5.4 Inclusion Criteria

#### 5.4.1 Stage A: Antenatal Care (ANC) Unit

The *first group* included high‐risk pregnant women who attended their ANC visit at the Volta regional hospital. It was necessary to have at least three ANC visits, and the survey needed to be done in the final trimester (> 28 weeks). Referred women were included only if they had attended at least two ANC visits at the Volta regional hospital before the last trimester survey. To identify high‐risk pregnancies, we used the criteria of Queenan, Spong [27], who define it as ‘any pregnancy with an unexpected medical or obstetric condition associated with the pregnancy or foetus posing a potential hazard to the health of the pregnant woman or foetus’. Women with high‐risk pregnancies were identified with the help of the ANC staff and the facility's registry. Selection criteria included pre‐gestational and gestational diabetes, severe maternal anaemia (haemoglobin < 7 g/dL), thrombophilia, sickle cell disease, placenta pre‐via, pre‐eclampsia, polyhydramnios, oligohydramnios, previous caesarean section, preterm ruptures of membranes (PROM), foetal anomaly and intrauterine growth restriction of the foetus and threatened preterm labour.

The *second group* comprised support persons of high‐risk pregnant women. These women visited at least one prenatal visit together, unrestricted by gender or age. If a high‐risk pregnant woman declined participation, her support person was still allowed to participate, and vice versa. Data collection was not conducted in pairs.

#### 5.4.2 Stage B: Labour Ward

The *third group* included high‐risk pregnant women around the time of birth. To ensure high‐risk pregnant women had sufficient experience with the labour ward, we surveyed women who had stayed in the ward for at least 12 h either before or after delivery. Data were collected before they were discharged home, at the latest 1 week after delivery. Mothers with planned caesarean sections were admitted directly to the postnatal care unit and were surveyed in the seventh group as they received care in the postnatal ward.

The *fourth group* included family members, without age restriction, who supported a pregnant woman during delivery.

#### 5.4.3 Stage C: Neonatal Intensive Care Unit

The *fifth and sixth groups* included mothers and family members of newborns admitted to the NICU within the neonatal period of < 28 days of life. ‘Small and sick newborns’ were defined as infants born prematurely (< 37 weeks of gestation), low birth weight (< 2500 g), early or late‐onset infections, respiratory distress, hyperbilirubinaemia, hypoglycaemia, birth anomalies or any other medical or surgical condition. In addition to the mother, any support person was eligible to take part in the survey as long as they had interacted with the HCPs at the time of the survey.

#### 5.4.4 Stage D: Postnatal Ward

The *seventh and eighth groups* of participants were mothers receiving care in the postnatal ward and their respective family members alongside newborns on the standard postnatal ward. Basic care for mothers and newborns is provided within the postnatal ward, alongside phototherapy when NICU capacity is restricted.

#### 5.4.5 Stage E: Community‐Based Primary Care

The *ninth group* included community participants. They were family members with a small or sick newborn and who had at least two contacts with HCPs at the Public Health and Nutrition Unit at the Volta regional hospital, the CHPS, health centre or received home visits at least twice within the last 8 weeks. The mother‐newborn dyads may have previously been admitted to a hospital other than the Volta regional hospital.

### 5.5 Study Sample

A sample size calculation was computed based on high‐risk pregnant women attending antenatal care. To achieve adequate representation across all levels of maternal and newborn care, for all eight other groups along the care continuum, sample sizes were estimated as outlined below, not on statistical considerations, but based on feasibility and expected participation rates, as informed by the literature described below.

The sample size for group 1, that is for the pregnant women at the ANC, was determined using the Cochran formula [28], where *N* stands for the desired sample size. $N=Z^{2}\mathrm{pq}/d^{2}$, *Z* = the desired confidence level (*α*), *P* = expected coverage, and *d* = the desired width of the confidence interval or precision. High‐risk pregnant women constitute 7.5% of all pregnant women [29]. Therefore, we set our sample size estimation on the following assumptions: prevalence of 7.5% = 0.075, reliability coefficient of 1.96 at 95% confidence level (CI) and 80% statistical power.

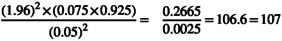




For group 2, the expected number of family members attending antenatal care (ANC) for high‐risk pregnancies was estimated using the 53% male involvement rate reported in Ghana [30]. Accordingly, 57 family members were expected to attend, based on 53% of the 107 high‐risk pregnancies.

For group 3 of birthing women, we expected 85 participants to receive high‐risk antenatal care (ANC), based on a 79% attendance rate reported by Abiiro, Gyan [30].

For group 4 of family members present in the labour ward, we used male attendance of 32% [30] and expected 34 participants.

For groups 5 and 7, it was expected that 31% of high‐risk pregnant women and their newborns would require postnatal inpatient care and NICU services, respectively, based on estimates from Appiah, Salihu [31], corresponding to 34 participants in each group.

For groups 6 and 8 of family members attending NICU and postnatal inpatient care, we used male involvement of 20% [32] and thus expected 22 participants in each.

For group 9 of mothers and family members in community‐based primary care, we included all participants reached between December 7, 2023 and January 30, 2024.

### 5.6 Measurement Instruments

To measure perceptions of support, we used the validated Iceland‐Family Perceived Support Questionnaire (ICE‐FPSQ) with permission from the developers [33]. The English version has been used in chronic and acute illnesses in various clinical settings worldwide, including South Africa [18], Nigeria [19] and Uganda [20].

The instrument consists of 14 items, divided into two subscales: cognitive support (5 statements) and emotional support (9 statements). The cognitive subscale measures HCPs' support in building family capacity for self‐reliance and empowerment. The emotional support subscale assesses the extent to which HCPs engage with families to allow open expression and acknowledgment of feelings associated with the illness experience [33].

The statements were measured on a 5‐stage Likert scale ranging from 1 (almost never) to 5 (almost always). The cognitive and emotional subscales have minimum and maximum scores of 5–25 and 9–45, respectively. The overall score ranges from 14 to 70 stages, with higher scores indicating perceptions of greater support. The ICE‐FPSQ has a well‐established Cronbach's *α* reliability coefficient of 0.96, with a cognitive subscale α = 0.88 and an emotional subscale *α* = 0.95 [33].

### 5.7 Variables

The primary dependent variables were the cognitive and emotional support scores. The independent variables included key socio‐demographic characteristics of each participant: age, gender and place of residence (urban or rural) and educational level as well as context variables such as type of participant, stage along the care continuum where care is provided, relationship with pregnant woman/newborn, information received and health facility attended. We also included the information provided by HCPs to these family members about the condition of high‐risk pregnant women and newborns.

### 5.8 Open‐Text Components

We added the following three open‐ended questions to the standardised questionnaire:

1. Was there anything you missed from the health professionals during your antenatal care, delivery, stay at the neonatal intensive care unit, postnatal care or the care provided at the community level?
2. Were there any other ways you or your family members would have liked to be involved in the care process during your pregnancy, labour, postnatal care at the NICU or ward or community care?
3. Is there anything else you think is important we did not ask?

The instrument was pretested in a different municipality in Ghana's Volta Region, involving family members with small or sick newborns. This pretesting allowed for the resolution of issues, such as accelerating responses through prompting of the research assistant.

We also made two minor context‐specific adjustments to the ICE‐FPSQ. For instance, the original sentence ‘The nurses on the unit have emphasised the use of family rituals to promote family members’ health… was adapted to ‘The healthcare professionals have emphasised the use of family routines (e.g., acts/prayers) to promote family members' health…’. Based on socio‐cultural contexts in Ghana, we used the term ‘healthcare professional’ instead of ‘nurse’ and changed ‘rituals’ to ‘routines’ to avoid confusion.

### 5.9 Data Collection

Three bilingual research assistants fluent in English and Ewe received data collection training, including a comprehensive overview of the research objectives and ethical considerations. Each question on the survey instruments and the socio‐demographics were discussed, and queries clarified during this training. Research assistants carried out a dummy data collection in a neighbouring municipality followed by constructive feedback. All questionnaires were personally administered by the research assistants. Participants completed the questionnaire in a private space while research assistants recorded their responses. This approach was adopted due to the varying literacy levels among the participants. Questions were asked in English, and where necessary, translated orally into Ewe. There was no follow‐up on participants after data collection.

### 5.10 Data Analysis

Data were analysed with STATA version 17/MP [34]. A univariate analysis was used for the socio‐demographics and is reported in frequencies and percentages. Cognitive, emotional and overall support were presented as means and standard deviations. Cronbach's alpha was calculated to assess the reliability of the ICE‐FPSQ overall scale and the subscales. We examined differences in support scores between participants' demographics (age group, gender, education level, employment status, residence and religion) and contexts (type of participant, stage in the care continuum, relationship with pregnant woman/newborn, information received and health facility attended) using the Student's *t*‐test for variables and an analysis of variance (ANOVA) for multi‐category variables, as appropriate. When ANOVA indicated significant differences, a Bonferroni post hoc test was conducted to identify the specific pair‐wise group differences. *p* values below 0.05 were considered statistically significant.

### 5.11 Analysis of Open‐Text Components

Open‐text components were analysed qualitatively in two cycles using NVivo 14 software. The first cycle involved open coding of the data and the second a thematic categorisation [35]. Divergent ideas were discussed among the researchers until a common understanding was reached.

### 5.12 Ethics Statement

This study was conducted in accordance with the principles of the Declaration of Helsinki, which emphasises confidentiality, integrity and the voluntary nature of participation [36]. We obtained ethical approval from the Ghana Health Service Ethical Review Committee and institutional permission from the Volta regional hospital management and the Hohoe Municipal Health Directorate. Information on the study was provided via an online link, and research assistants read it aloud to participants who could not read English to obtain verbal consent from all participants. Adult guardians confirmed consent for participants below 18 years of age. All participants were informed of their rights, including the right to withdraw at any stage without consequences. Confidentiality was maintained throughout the study; no names or contacts were recorded, and analyses were carried out anonymously.

## 6 Results

### 6.1 Participants

A total of 442 participants were included from the five stages across the care continuum (Table 1). Most were female (90%) with a mean age of 32.2 years; 55% lived in urban areas. Among the participants, 40% had attended junior high school, 17% had received tertiary education, and 75% were employed.

**TABLE 1:** Participants' socio‐demographics compared along the different continuum of care.

Stages in the care continuum	Stage A antenatal care		Stage B labour ward		Stage C neonatal intensive care unit		Stage D postnatal care unit		Stage E community‐based primary care	
Characteristic	Group 1	Group 2	Group 3	Group 4	Group 5	Group 6	Group 7	Group 8	Group 9	Total
	Pregnant woman	Family members	Birthing women	Family members	Mothers	Family members	Mothers	Family members	Mothers	
	*n* = 140	*n* = 29	*n* = 53	*n* = 35	*n* = 44	*n* = 21	*n* = 37	*n* = 32	*n* = 51	*N* = 442
	*n* (%)	*n* (%)	*n* (%)	*n* (%)	*n* (%)	*n* (%)	*n* (%)	*n* (%)	*n* (%)	*n* (%)
Gender										
Female	140 (100.0)	12 (41.4)	53 (100.0)	24 (68.6)	44 (100.0)	14 (66.7)	37 (100.0)	26 (81.3)	51 (100.0)	401 (90.7)
Male	—	17 (58.6)	—	11 (31.4)	—	7 (33.3)	—	6 (18.7)		41 (9.3)
Age (years) mean (SD)	28.9 ± 6.5	36.7 ± 9.5	30.0 ± 10.0	37.8 ± 13.7	28.1 ± 10.1	40.9 ± 14.2	32.1 ± 11.2	44.1 ± 13.8	29.6 ± 7.4	32.2 ± 10.9
< 20^a^	17 (12.1)	6 (20.7)	5 (9.4)	1 (2.9)	11 (25.0)	1 (4.8)	3 (8.1)	—	2 (3.9)	40 (9.0)
20–29	52 (37.1)	12 (41.4)	20 (37.7)	11 (31.4)	14 (31.8)	3 (14.3)	14 (37.8)	6 (18.8)	25 (49.0)	151 (34.2)
30–39	65 (46.4)	8 (27.6)	23 (43.4)	10 (28.6)	15 (34.1)	7 (33.3)	12 (32.4)	5 (15.6)	22 (43.1)	171 (38.7)
40–49	6 (4.3)	3 (10.3)	4 (7.5)	7 (20.0)	3 (6.8)	4 (19.0)	7 (18.9)	9 (28.1)	1 (2.0)	49 (11.1)
50+	—	3 (10.3)	1 (1.9)	6 (17.1)	1 (2.3)	6 (28.6)	1 (2.7)	12 (37.5)	1 (2.0)	31 (7.0)
Education										
None	11 (7.9)	3 (10.3)	6 (11.3)	3 (8.6)	2 (4.5)	3 (14.3)	1 (2.7)	9 (28.1)	3 (5.9)	41 (9.3)
Primary	8 (5.7)	2 (6.9)	8 (15.1)	4 (11.4)	4 (9.1)	3 (14.3)	6 (16.2)	7 (21.9)	3 (5.9)	45 (10.2)
Junior High School	56 (40.0)	10 (34.5)	23 (43.4)	16 (45.7)	22 (50.0)	5 (23.8)	20 (54.1)	6 (18.7)	20 (39.2)	178 (40.3)
Senior High School	36 (25.7)	4 (13.8)	11 (20.8)	10 (28.6)	4 (9.1)	6 (28.6)	7 (18.9)	7 (21.9)	16 (31.4)	101 (22.8)
Tertiary	29 (20.7)	10 (34.5)	5 (9.4)	2 (5.7)	12 (27.3)	4 (19.0)	3 (8.1)	3 (9.4)	9 (17.6)	77 (17.4)
Employment status										
Employed	109 (77.9)	23 (79.3)	36 (67.9)	28 (80.0)	31 (70.5)	18 (85.7)	31 (83.8)	26 (81.3)	31 (60.8)	333 (75.3)
Unemployed	31 (22.1)	6 (20.7)	17 (32.1)	7 (20.0)	13 (29.5)	3 (14.3)	6 (16.2)	6 (18.7)	20 (39.2)	109 (24.7)
Residence										
Rural	38 (27.1)	6 (20.7)	33 (62.3)	19 (54.3)	16 (36.4)	10 (47.7)	21 (56.8)	19 (59.4)	34 (66.7)	196 (44.3)
Urban	102 (72.9)	23 (79.3)	20 (37.7)	16 (45.7)	28 (63.6)	11 (52.3)	16 (42.2)	13 (40.6)	17 (33.3)	246 (55.7)
Religion										
Christian	115 (82.1)	27 (93.1)	46 (86.8)	28 (80.0)	41 (93.2)	19 (90.6)	31 (83.8)	25 (78.1)	43 (84.3)	375 (84.8)
Muslim	22 (15.7)	2 (6.9)	6 (11.3)	4 (11.4)	3 (6.8)	1 (4.7)	6 (16.2)	7 (21.9)	8 (15.7)	59 (13.4)
Traditional	3 (2.1)	—	1 (1.9)	3 (8.6)	—	1 (4.7)	—	—	—	8 (1.8)
Relation to pregnant woman^b^										
Husband	—	14 (48.3)	—	8 (22.9)	—	—	—	—	—	22 (34.4)
Other relations	—	15 (51.7)	—	27 (77.1)	—	—	—	—	—	42 (65.6)
Relation to baby^c^										
Mother	—	—	—	—	44 (100.0)	—	37 (100.0)	—	50 (98.0)	131 (70.8)
Father	—	—	—	—	—	7 (33.3)	—	6 (18.7)	—	13 (7.0)
Other relations	—	—	—	—	—	14 (66.7)	—	26 (81.3)	1 (2.0)	41 (22.2)

^a^ Between age 14 and 17. Group 1: *n* = 7, Group 3: *n* = 4, Group 5: *n* = 6, Group 7: *n* = 2, Group 9: *n* = 1.

^b^ Answered by Group 2, Group 4.

^c^ Answered by Group 5, Group 6, Group 7, Group 8.

Of the 64 family members of the pregnant and birthing women, 34.4% (*n* = 22) were husbands, whereas the remaining 65.6% (*n* = 42) family members included mothers, sisters, sisters‐in‐law, grandmothers and fathers. Among the 185 participants catering for newborns, 70.8% (*n* = 131) were mothers, 7.0% (*n* = 13) fathers and other family members 22.2% (*n* = 41) including grandmothers, aunties, cousins, and neighbours. No family members actively participated in community‐based primary care visits.

### 6.2 Support Perception Scores

Table 2 details overall and subscales of families' perception of support. The mean overall score was 45.3 (SD = 14.2) of a maximum of 70. Cognitive support recorded 16.6 (SD = 5.9) of 25. The highest‐rated item was the statement on ‘The healthcare professionals have offered us information and their professional opinion’ and the lowest‐rated statement was ‘The healthcare professionals informed my family about the resources available in the community that helped families in similar situations’.

**TABLE 2:** Mean scores obtained for cognitive and emotional support (*n* = 442).

Statements	Mean (SD)
Cognitive support (CS) Subscale (Min. 5–Max. 25)	16.6 (±5.9)
**CS1**—The healthcare professionals have offered us information and their professional opinion	3.9 (±1.4)
**CS2**—The healthcare professionals provided accessible and easy‐to‐read literature or showed us videos about the health problem of our family member (small/sick baby)	3.2 (±1.8)
**CS3**—The healthcare professionals informed my family about the resources available in the community that helped families in similar situations	2.2 (±1.6)
**CS4**—The healthcare professionals provided ideas, information and thoughts in a manner which allowed us to learn from them and think about them	3.9 (±1.4)
**CS5**—The healthcare professionals have emphasised the use of family routines (e.g., acts/prayers) to promote family members' health	3.4 (±1.8)
Emotional support (ES) subscale (Min. 9–Max. 45)	28.6 (±10.1)
**ES1—**The healthcare professionals offered us family discussions	2.8 (±1.9)
**ES2—**The healthcare professionals have helped my family members recognise that our emotional response is acceptable and helped us to perceive and validate/normalise the emotional response of family members	3.5 (±1.7)
**ES3—**The healthcare professionals have encouraged my family to become involved with the healthcare team in the care of our family member (baby) and have offered us caregiver support	3.4 (±1.7)
**ES4—**The healthcare professionals have encouraged my family members to share their illness narratives—not only stories of illnesses and suffering, but also stories of strength and resilience	2.5 (±1.8)
**ES5—**The healthcare professionals have drawn out our family strengths	2.9 (±1.5)
**ES6—**The healthcare professionals have helped family members understand how our emotional response is related to the family member's (baby's) illness	3.3 (±1.8)
**ES7—**The healthcare professionals have encouraged my family to help me take a respite/break from daily chores sometimes	4.0 (±1.6)
**ES8—**The healthcare professionals understand how family members affect one another, the baby's health/well‐being and also the illness itself	3.5 (±1.4)
**ES9—**The healthcare professionals looked for the family's strengths and opportunities to commend family members when their strengths have been revealed	2.7 (±1.7)
Overall score ICE‐FPSQ (Min. 14–Max. 70)	45.3 (±14.2)

*Note:* ICE‐FPSQ: Iceland‐Family Perceived Support Questionnaire, high score, high perceived support.

Emotional support had a mean score of 28.6 (SD = 10.1) of a maximum of 45. The highest‐rated item was ‘The healthcare professionals encourage my family to help me take a respite/break from daily chores sometimes’ and the lowest‐rated item was ‘The healthcare professionals have encouraged my family members to share their illness narratives – not only stories of illnesses and suffering but also stories of strength and resilience’.

### 6.3 Influence of Demographic Characteristics on Family Support

As detailed in Table 3, females (16.8 ± 5.8), Muslims (18.1 ± 4.8), city and town residents (17.7 ± 5.6) and participants with higher education and employment (17.1 ± 5.8) scored higher in cognitive support than their counterparts. Older participants (≥ 50 years) reported significantly lower cognitive support scores (11.4 ± 5.2). Support scores differed across the care continuum and between relationship categories. Pregnant women at ANC scored highest for cognitive support (20.2 ± 4.1), and mothers with small/sick newborns at the postnatal care for emotional support (33.7 ± 9.2). Before delivery (ANC), husbands of pregnant women received higher emotional support (32.8 ± 10.1) than other relatives (25.8 ± 10.8). After birth, mothers (15.9 ± 5.5) and fathers (15.7 ± 6.2) reported higher cognitive support than family members (12.1 ± 5.8). Family members in the NICU obtained the lowest overall score across all domains (36.6 ± 12.7), followed by mothers at the community‐based primary care level (38.0 ± 12.5). Information provision concerning high‐risk pregnancy is possibly associated with cognitive (19.8 ± 4.3) but not with emotional support.

**TABLE 3:** Association between perceived support and independent variables.

Characteristic	Cognitive support		Emotional support		Total perceived support	
	Mean (SD)	*p*	Mean (SD)	*p*	Mean (SD)	*p*
Gender^a^		0.0432		0.4258		0.7854
Female	16.8 (5.8)		28.5 (9.9)		45.3 (14.0)	
Male	14.9 (6.2)		29.8 (11.2)		44.7 (16.1)	
Age^b^		< 0.0001		0.5444		0.1118
14–20	16.9 (4.9)		28.4 (9.7)		45.2 (12.6)	
20–29	16.7 (5.8)		28.0 (9.6)		44.7 (13.7)	
30–39	17.5 (5.6)		28.8 (10.1)		46.3 (13.8)	
40–49	16.6 (6.7)		30.8 (11.1)		47.4 (16.8)	
50+	11.4 (5.2)		28.0 (10.7)		39.4 (14.8)	
Education^b^		0.0285		0.8213		0.4414
None	14.4 (6.7)		26.9 (11.6)		41.4 (17.5)	
Primary	16.0 (6.2)		29.2 (10.3)		45.2 (15.3)	
JHS	16.7 (5.5)		29.0 (9.9)		45.7 (13.7)	
SHS	16.6 (5.8)		28.7 (9.9)		45.3 (13.8)	
Tertiary	18.1 (5.7)		28.4 (9.9)		46.4 (13.0)	
Employment^a^		0.0029		0.0228		0.0043
Employed	17.1 (5.8)		29.3 (9.9)		46.4 (13.9)	
Unemployed	15.2 (5.9)		26.7 (10.3)		41.9 (14.7)	
Residence^b^		< 0.0001*		0.4783		0.0269*
Rural	15.4 (5.8)		28.2 (9.9)		43.6 (14.2)	
Urban	17.7 (5.6)		28.9 (10.2)		46.6 (14.0)	
Religion^b^		0.0073		0.0213		0.0068
Christian	16.5 (6.0)		28.2 (10.2)		44.7 (14.4)	
Muslim	18.1 (4.8)		31.8 (8.8)		49.9 (12.0)	
Traditional	11.5 (5.8)		25.0 (5.6)		36.5 (9.9)	
Population^b^		< 0.0001		0.0010		< 0.0001
Pregnant women at ANC	20.2 (4.1)		28.8 (9.0)		49.0 (10.9)	
Family members at ANC	15.0 (6.0)		29.0 (12.0)		44.0 (17.0)	
Mothers at labour ward	16.6 (5.7)		31.1 (10.9)		47.6 (15.9)	
Family members at labour ward	12.2 (5.7)		27.5 (10.2)		39.7 (14.6)	
Mothers at NICU	16.8 (5.7)		28.1 (9.9)		45.0 (13.0)	
Family members at NICU	12.1 (5.3)		24.5 (8.8)		36.6 (12.7)	
Mothers at postnatal ward	18.5 (5.0)		33.7 (9.2)		52.2 (12.9)	
Family members at postnatal ward	13.3 (6.6)		28.6 (11.9)		41.9 (17.4)	
Primary care level	13.4 (4.6)		24.5 (8.9)		38.0 (12.5)	
Relation to pregnant woman^a^		0.1440		0.0135		0.0231
Husband	14.9 (6.1)		32.8 (10.1)		47.8 (15.0)	
Other relations	12.7 (5.7)		25.8 (10.8)		38.4 (15.4)	
Relation to baby^b^		0.0008		0.6235		0.0998
Mother	15.9 (5.5)		28.2 (10.0)		44.2 (14.9)	
Father	15.7 (6.2)		29.1 (11.2)		44.8 (16.1)	
Other relations	12.1 (5.8)		26.6 (10.8)		38.7 (15.7)	
Informed about high‐risk pregnancy^b^		< 0.0001		0.8377		0.0642
No	16.1 (6.2)		29.2 (10.8)		45.2 (16.0)	
Yes	19.8 (4.3)		29.3 (8.7)		49.1 (9.9)	
Not sure/cannot remember	16.7 (5.4)		27.5 (11.5)		44.2 (15.3)	
Given information for admission to NICU^b^		0.0061		0.1426		0.0366
No, no specific details given	11.7 (5.6)		24.4 (9.4)		36.1 (13.4)	
We received partial information	15.3 (6.3)		29.9 (11.9)		45.2 (17.7)	
Yes, we were provided with clear information	15.7 (5.6)		28.2 (10.0)		43.9 (14.0)	
Health facility attended^b^		0.6280		0.5764		0.5442
CHPS compound	12.8 (4.2)		23.2 (8.1)		36.0 (11.6)	
Health centre	13.7 (5.2)		25.5 (11.5)		39.2 (14.1)	
Public health unit and Volta regional hospital	14.3 (5.0)		26.0 (8.7)		40.3 (13.1)	

^a^ *p* value from *t*‐test.

^b^ *p* value from ANOVA.

### 6.4 Internal Reliability of the ICE‐FPSQ Scale

The overall score of the ICE‐FPSQ scale had a Cronbach's α of 0.87, the cognitive support subscale with an α of 0.78 and the emotional support subscale of an α of 0.84. There were no missing data.

### 6.5 Findings From Open‐Text Components

A total of 168 responses from 130 participants to the open‐ended questions were recorded. Open‐text components were classified as (1) opportunities for family involvement with two sub‐categories and (2) missed care opportunities with four sub‐categories.

#### 6.5.1 Opportunities for Family Involvement Along the Care Continuum

##### 6.5.1.1 Family Involvement in Care Provision

Women expressed the wish for family members to be involved in care procedures and decision‐making: ‘The family should get involved in any decision concerning my well‐being as a pregnant woman’. (Pregnant woman, 36 years, antenatal clinic).

Families wished to be well informed about the newborns' health: ‘I would have loved to be more involved with the progress report of our baby's health’. (Aunt, 30 years, NICU).

Women receiving care at different stages of the care continuum wanted other family members to be around to support them. This request was especially prominent for the period of labour or when their newborn was admitted to the NICU: ‘The family would want a comfortable place to be provided for them to sleep near their relatives so they can assist them in the night’. (Mother of the birthing woman, 69 years, labour ward).

Other family members mentioned not being given sufficient time for visits, although their presence could serve as an encouragement: ‘Family members should be allowed enough time for visits’. (Pregnant woman, 40 years, antenatal clinic) or ‘We want to be around and encourage her’. (Sister, 29 years, labour ward).

##### 6.5.1.2 Acceptance of Alternative Medicine and Religious Faith

The attitudes and traditional perspectives of family members towards traditional/herbal medicine were apparent in their responses to the open questions throughout the care continuum: ‘Family members should be allowed to use herbal medicines for our relatives on admission at the hospital’. (Husband, 38 years, antenatal clinic) or ‘I would like to give her herbal medicine to help her deliver fast’. (Mother of the pregnant woman, 45 years, labour ward).

They also wanted to use herbal medicine to treat the newborn: ‘We would want to use herbs for the swollen arm (of the baby)’. (Mother, 23 years, NICU).

Family members also held the belief that prayers served as a means of facilitating the healing of the newborn: ‘They should allow us to go and pray for our sick babies’. (Father, 40 years, NICU).

#### 6.5.2 Missed Opportunities Along the Care Continuum

##### 6.5.2.1 Long Waiting Times

Pregnant women reported long waiting times at the antenatal care unit, with husbands and other family members expressing frustration over the delays when accompanying their female relatives to the hospital: ‘Too much time is spent (at the hospital) when I bring my wife’. (Husband, 37 years old, antenatal clinic).

Similarly, obtaining laboratory and imaging results was frequently reported as time‐consuming, with some patients never receiving their results: ‘I did not get to see my lab results on time and sometimes I did not even see them at all’. (Pregnant, 30 years old, antenatal clinic).

##### 6.5.2.2 Dissatisfied Engagement Between Healthcare Providers and Family Members

A lack of attention and empathy was predominantly reported during the birthing period: ‘The health workers did not pay attention to my complaints, and they don't have empathy for us when we are in pain’. (Pregnant woman, 33 years old, labour ward).

Pregnant women complained about minimal support from HCPs, while they may have received it from the family: ‘I didn't have any family around (during birth), but they (healthcare professionals) did not assist me to bathe’. (Mother, 38 years old, postnatal care unit).

Mothers expressed a desire for mandatory male involvement throughout the various stages of care, as the presence of their partners provided both financial and emotional support: ‘I would have preferred that healthcare professionals make it a must for our husbands to always be taking us to the hospital’. (Mother, 34 years old, primary care).

Family members and women wished HCPs would be more patient during interactions at the various levels of care. They were often dissatisfied with the way HCPs communicated: ‘Some of the healthcare professionals don't talk to us politely’. (Mother, 24 years old, postnatal care unit) or ‘The healthcare professionals should be patient with me’. (Mother, 32 years old, CHPS zone).

They also expressed the desire for HCPs to listen to their concerns. They criticised them for not adequately acknowledging and addressing their needs, and they expressed concerns, attributing adverse neonatal health outcomes to a lack of knowledge: ‘They were not paying attention to my sister when she was in labour. That led to the baby's condition’. (Sister, 29 years old, labour ward).

##### 6.5.2.3 Delayed Breastfeeding

Mothers reported long delays before seeing their newborns after birth, which they felt led to delayed breastfeeding and ultimately engorged breasts. They reported insufficient beds for mothers with babies in the NICU as a significant obstacle: ‘I will be happy if the mothers at NICU are provided with facilities so that we can stay and take care of our babies’. (Mother, 40 years old, NICU) or ‘There should be accommodation provided for mothers at the NICU’. (Father, 40 years, NICU).

Family members presumed the delays in breastfeeding had led to complications: ‘They didn't allow my daughter to breastfeed the child early, so that led to the jaundice’. (Grandmother, 44 years, NICU).

##### 6.5.2.4 Insufficient Health Education and Information on Care Procedures

Pregnant women expressed a desire for additional educational sessions on pregnancy, childbirth and sexual health after caesarean section and emphasised the importance of including other family members in these information sessions: ‘More education should be given about pregnancy and childbirth’. (Pregnant woman, 33 years old, antenatal clinic) or ‘The family should be encouraged to come to ANC and listen to health education’. (Pregnant woman, 36 years old, antenatal clinic). They also wished instructions were given with better explanations: ‘I want to know why they (HCPs) always asked us to sleep on their left side but not our right side’. (Pregnant woman, 32 years old, antenatal clinic) or ‘They should explain the procedure well before we have to go to the theatre (caesarean section)’. (Mother, 38 years old, primary care).

Surgical procedures and laboratory tests were described with inadequate clarity to women and families desiring to receive thorough information, especially about the baby's health.

‘They were not as expressive as they should be in giving us information about the health status of our baby’. (Aunt, 30 years old, NICU).

Families cited receiving insufficient education from HCPs and a lack of encouragement, which negatively impacted confidence‐building. ‘Lack of education and encouragement to help build our confidence’. (Mother, 28 years old, primary care).

## 7 Discussion

Overall, support by HCPs throughout the continuum of care scored average on the 5‐point Likert scale, suggesting that while some support was present, it may not have fully aligned with the needs of families.

Cognitive support received higher ratings than emotional support. Several factors correlated with higher cognitive support, in particular young age, pregnant women, mothers or fathers of the newborn, urban residence, being Muslim and provision of high‐risk pregnancy information and the reason for NICU admission. Higher emotional support was reported for husbands of pregnant women, Muslims, and when the reason for admission of the newborns was communicated clearly.

The primary criticisms by family members were their exclusion from the care plans and the absence of male involvement. Families also expressed their desire to be listened to and the acceptance of alternative medicine and spirituality.

Cognitive support was rated higher than in the previous African studies. As detailed below, higher cognitive support was associated with younger age, pregnant women, mothers, fathers, Muslims and urban residents, indicating that HCPs allocate more support to these groups. Cognitive support scores were significantly lower among individuals aged 50 and above, despite their crucial role in caring for women and newborns in Africa. This disparity raises concerns about the lack of information that these individuals genuinely desire. Emmamally and Brysiewicz [18] reported similar findings where older family members perceived HCP support as low compared to other age groups. We believe that the involvement of older relatives, such as grandmothers, is generally beneficial for both the woman and the child [37]. However, the index person (typically the mother) should always be asked whom she wishes to have involved in her or her newborn's care [15].

We found that awareness of community resources was low, aligning with findings from Nigeria [19] but contrasting with a study in Uganda [20]. Healthcare professionals may have inadvertently omitted discussions about community resources because of time constraints, a desire to avoid community stigma and preconceived notions associated with preterm newborns might be other reasons [5]. The active translation of standardised guidelines of FSC and continuity of care into practice would facilitate the connection between families and community resources [38, 39]. Consequently, this gap may be effectively bridged.

Participants rated ‘Healthcare professionals provided information and opinions’ highest on the cognitive support subscale, mirroring findings from the Nigeria study on adult care [19]. In South Africa, families gave lower scores for information received [18], suggesting obstacles to delivering family‐focused care in high‐acuity settings such as emergency rooms, where the urgent demands of acute care can limit the provision of information. Despite higher scores, open‐text components in our study indicated that families require more comprehensive information and educational resources throughout the care continuum.

Mothers who received information about their own high‐risk pregnancy or their child's illness had higher cognitive scores, but not overall scores. In contrast to the findings of Sigurdardottir, Garwick [40], mothers who received information about their child's illness exhibited higher overall scores. This difference can only be explained by lower emotional support scores, which we will discuss below. Detailed explanation and information on maternal and newborn health, illness or management is essential [41, 42]. Information and explanations help families understand and prepare for what comes next.

Emotional support scored lower than cognitive support but was higher than in Nigeria and South Africa [18, 19]. Low levels of emotional support were also evident from responses to open‐ended text components. Notably, the lowest emotional support was found in the NICU, where emotional and psychological support is often needed the most after having a preterm or sick newborn [43]. Our data cannot definitively ascertain whether low emotional support stems from an unwelcoming environment, a lack of privacy, empathy or inconsiderate communication. However, unfavourable communication was indeed reported and believed to hinder the willingness to share stories about their illnesses, resilience and achievements.

The infrequent invitations by HCPs to involve families in discussions may have contributed to the low emotional support scores. While we found higher family discussion scores than in the emergency units [18], acute care settings exhibited higher scores [19, 20]. This difference might be due to the complexity of planning family discussions influenced by factors such as HCPs' attitudes, substantial workloads and patients' medical conditions. Adequate staffing and space for family discussions may increase the likelihood of such discussions.

Low levels of sharing illness narratives were also reported in two other African studies conducted in hospital and community settings [19, 21]. Yet, sharing such narratives is recognised as an important first step toward stress reduction and healing for patients and their families [44].

The emotional item ‘identifying family strengths and commending family members for them’ had a low score. Since familial awareness of inherent strengths is crucial for facilitating access to resources and information [40], it may serve as a key target for recommendations. Noteworthy in our study, husbands reported higher emotional support when information about the reason for the newborn's admission was communicated clearly.

A striking difference between our findings and those published in Uganda [20] was found for the emotional item respite and rest. We believe this may originate in a traditional perception of the importance of postpartum rest for mothers in Ghana [45]. HCPs should consider factors like the severity of the illness, work commitments and family members' individual need for rest before recommending respite from care [15]. In Ghana and similar contexts, the existence of extended family networks can offer assistance, thereby enabling rest. Such assistance is particularly important for women in the perinatal stage, with complicated pregnancies or birth experiences, or having their infant admitted to the NICU [42, 46].

In maternal and neonatal care in low‐ and middle‐income countries, essential nursing functions, encompassing education, emotional support and communication with families are often neglected [41, 47]. This neglect mirrors our low emotional support scores. Important emotional support interventions, such as active relational listening and therapeutic questioning [44, 48], may be frequently absent from HCPs' training and in practical placements in Ghana and other African countries [42, 49]. But cognitive and emotional support foster the relationship between HCPs and families, ultimately facilitating the workflow.

The participants' main complaint concerned the lack of family member inclusion, especially of men. In Ghana, male participation in maternal care has recently been reported at 52% [30]. Our findings show that many women and some men favour greater male involvement in maternal and newborn care, with some supporting its mandatory inclusion. This highlights the need for couple‐focused interventions and community sensitisation. Low family involvement, however, is hindered by long wait times, travel costs, limited ward space and privacy concerns—factors that also explain difficulties in recruiting family members, especially in ANC, labour wards and community health settings.

Families of Muslim faith demonstrated higher levels of perceived support compared to Christian and Traditional believers across all the scores and sub‐scores. Muslims consider caring for themselves, family, friends, and neighbours to be an act of worship [50]. We thus hypothesise that families of the Muslim faith tend to advocate for their ill relatives to a greater extent than families of other belief systems and thus receive more support from HCPs.

## 8 Strength and Limitations

Strengths of this study are its coverage of the entire continuum of maternal and neonatal care, capturing perspectives from pregnancy through community follow‐up, a comprehensive approach that few studies, if any, have taken. The substantial sample size of 442 participants, encompassing a diverse range of family members, enhances the robustness of the conclusions and using a validated questionnaire (ICE‐FPSQ) allows quantitative benchmarking of perceived support. The high reliability, as shown by a Cronbach's α of 0.87, confirms the tool's applicability to our context. Finally, the addition of open‐text questions enriches the context‐specific findings.

A limitation of the study may be reduced responsiveness from mothers, given the busy nature of the childbirth and postnatal period and possible fatigue. Participants did not self‐apply the questionnaires, and most surveys were completed within the hospital, making them prone to social desirability bias. A potential limitation was the population's low literacy level, especially among women. Some participants may have felt uncomfortable during the survey, even though it was administered in the local language, and may have felt unable to refuse participation or give full responses to the open‐text questions.

Participants were recruited from one single municipality, which may decrease the generalisability of results. Methodological considerations include the absence of a defined threshold in the questionnaire for distinguishing adequate from inadequate support [51]. The primary sample size was calculated for high‐risk pregnant women, whereas sizes for other groups were based on feasibility, potentially reducing estimate precision. We assumed simple random sampling without adjustment for potential clustering effects within healthcare facilities or non‐response rates.

## 9 Implications for Practice and Further Research

Family systems care guidelines and checklists can be used to guide HCPs in their daily practice. Healthcare delivery would be enhanced by implementing a stricter agenda with scheduled short discussion times to reduce waiting and by providing timely updates to families during delays, which may improve perceptions of support. Across the perinatal care continuum, care can be strengthened by scheduling designated family meetings, using checklists to ensure cognitive and emotional support, and actively involving family in perinatal care. These efforts could be reinforced through community outreach, in alignment with cultural norms.

Future qualitative research could allow for a more detailed examination of the quantitative findings, with an emphasis on the viewpoints of families and healthcare professionals. A possible hypothesis emerging from these findings is that communication‐skills training increases HCPs' ability to provide emotionally safe care.

## 10 Conclusion

Families perceived the overall support from HCPs along the care continuum as low and clearly lower for emotional than cognitive support. Healthcare delivery is likely to be enhanced by organisational changes, such as implementing a stricter agenda with scheduled short discussion times to reduce waiting, and by providing timely updates to families during delays, which can improve perceptions of support. Comprehensive family support that addresses both cognitive and emotional needs requires adequate healthcare personnel to provide daily care. Infrastructure improvements are needed to ensure privacy for mothers and newborns while accommodating family members, for example, through temporary lodgings.

Scheduling family meetings and using guidelines and checklists can improve family resilience and involvement through the provision of emotional and cognitive support within the scope of perinatal care. These efforts could be reinforced through community outreach, in alignment with cultural norms. To improve the overall care experience and its quality, easy‐to‐understand information on illnesses should be provided in a timely manner throughout the perinatal period to all family members, including male partners and older relatives. This requires HCPs to receive education on providing emotional support and effective relational communication, as well as technical knowledge of risks and benefits of traditional and herbal treatments. The application of in‐service training and training curricula for healthcare professionals, with a focus on family systemic approaches to skill enhancement in cognitive and emotional support, is vital for ensuring high‐quality, clinically secure and psychologically and emotionally safe perinatal care.

## Author Contributions

Conceptualization: C.S., A.F., B.P.‐B., R.E.P. Data curation: C.S., W.K.A. Formal analysis: W.K.A., G.E.N., C.S. Funding acquisition: C.S. Investigation: C.B., C.S. Methodology: C.S., W.K.A., G.E.N. Project administration: C.S. Resources: C.S. Software: W.K.A., G.E.N., C.S. Supervision: A.F., B.P.‐B., R.E.P. Validation: C.S., A.F., R.E.P. Visualization: W.K.A., C.S., G.E.N. Writing – original draft: C.S., G.E.N. Writing – review and editing: C.S., G.E.N., E.B., W.K.A., R.E.P., B.P.‐B., A.F.

## Funding

The University of Geneva provided financial support for the publication of this article.

## Ethics Statement

Ethical approval was granted by the Ghana Health Service Ethical Review Committee (GHS ERC) ID Nr: GHS‐ERC 027/03/23 on July 8th, 2023. The study was approved by the hospital management and the district health directorate of the study location.

## Consent

Participants gave verbal informed consent to participate in this study after the study information was read to them.

## Conflicts of Interest

The authors declare no conflicts of interest.

## Data Availability

The data that support the findings of this study are available from the corresponding author upon reasonable request.
